# DSCR9 gene simultaneous expression in placental, testicular and renal tissues from baboon (*papio hamadryas*)

**DOI:** 10.1186/1756-0500-5-298

**Published:** 2012-06-15

**Authors:** Irám Pablo Rodriguez-Sanchez, María Lourdes Garza-Rodríguez, María Elizabeth Tejero, Shelley A Cole, Anthony G Comuzzie, Hugo Alberto Barrera-Saldaña

**Affiliations:** 1 Vitaxentrum. Blvd. Puerta del Sol 1005, Colinas de San Jerónimo, Monterrey, Nuevo León, 64460, Mexico; 2 Facultad de Medicina, Departamento de Bioquímica y Medicina Molecular, Universidad Autonoma de Nuevo León, Av. Madero y Dr. Aguirre Pequeño, Col. Mitras Centro, Monterrey, Nuevo León, 64460, Mexico; 3 Auxology and Metabolism Working Group, and Texas Biomedical Research Institute, San Antonio, TX, USA; 4 Instituto Nacional de Medicina Genómica, Periférico sur 4124. Torre Zafiro II Col. Ex Rancho de Anzaldo, México, DF, Mexico; 5 Department of Genetics, Texas Biomedical Research Institute, San Antonio, TX, USA

**Keywords:** DSCR, Primate, Gene expression

## Abstract

**Background:**

In 2002 Takamatsu and co-workers described the human *DSCR9* gene and observed that it was transcriptionally active in human testicular tissue, but no protein was identified as a product of this transcript. Similar results were obtained in chimpanzee tissue. This gene has not been detected in species other than primates, suggesting that *DSCR9* is exclusively found in these mammals.

**Results:**

We report evidence of *DSCR9* expression in placenta, testis and kidney of baboon (*Papio hamadryas*). We used primers specific for *DSCR9* to amplify transcripts through reverse transcription (RT) coupled to polymerase chain reaction (PCR). Furthermore, PCR was used to amplify the complete *DSCR9* gene from genomic DNA from three baboons. We amplified and sequenced five overlapping segments that were assembled into the 3284 bp baboon *DSCR9* gene, including the putative promoter and the entire transcriptional unit (5'-UTR, CDS and 3'-UTR).

**Conclusions:**

The baboon *DSCR9* gene is highly similar to the human counterpart. The isolated transcripts from baboon tissues (placenta, testis and kidney) of three different baboons correspond to the human orthologous gene.

## Background

Down syndrome (DS) or trisomy 21 is the most common chromosome disorder affecting newborns and the most frequent and recognized cause of mental retardation in *Homo sapiens* (Hosa)[1]. The incidence of this syndrome is about 1 in 700 newborns [2]. Chromosome 21 is the smallest of human autosomal chromosomes, and an extra copy or additional segment of this chromosome causes DS [3]. The chromosomal region responsible for this pathology has been described [4] and named Down Syndrome Critical Region (DSCR)[5,6]. By comparing the genomic DSCR sequence in humans with that of other species, it was shown that it is highly conserved in great apes[6] and similar trisomies have been described in these non human primates [7,8]. In humans, ten potential genes have been identified in the DSCR, two of which (*DSCR9* and *DSCR10*) are exclusive of primates [9]. In man, *DSCR9* gene transcription, but not proteins, were evidenced in testicle; this was also demonstrated in chimpanzee [9]. The aim of this study was to identify the chromosome segment from which the *DSCR9* gene´s transcripts originated.

We amplified five segments from the baboon genome using primers designed to render overlapping amplicons. Using this strategy we were able to assemble the complete *DSCR9* gene of the species. The isolated genomic segment includes the putative promoter and the complete transcriptional unit. Using reverse transcription (RT) coupled to polymerase chain reaction (PCR), we have gathered evidenced of the expression of the *DSCR9* gene in baboon’s placenta, testicles, and kidney, an of its lack of detectable expression in heart, omental fat, skeletal muscle, pancreas, mononuclear cells, liver, and hypothalamus. The identified transcripts of these tissues in three different individual were identical and correspond to the isolated *DSCR9* baboon gene.

## Methods

### Animal specimens

Animal procedures were performed according to ethical guidelines and reviewed by the Institutional Animal Care and Use Committee of the Texas Biomedical Research Institute (TBRI). Animals were maintained at the Southwest National Primate Research Center in San Antonio, Texas at TBRI. All the animals shared the same diet and environmental conditions before and during pregnancy. All baboons are gang-housed and fed *ad libitum* on a standard low-fat chow diet (Harlan Tecklad 15% Monkey Diet, 8715).

### Biological samples

Different tissues from tree male baboons (testis, kidney, heart, omental fat, skeletal muscle, pancreas, mononuclear cells, liver, and hypothalamus) and from tree female baboons (ovary and placenta) were collected in programmed necropsies, under fasting conditions. Placental tissues were collected by caesarean section at the time of birth [at the term period of gestation for this species (136 to 139 days)]. All tissues were stored in liquid nitrogen immediately after collection until needed.

### Nucleic acid isolation from placental tissue

Genomic DNA and total RNA were isolated from each tissue using TRIZOL reagent; procedures were performed according to the manufacturer's instructions (Invitrogen, Carlsbad, CA). RNA samples were treated with DNase I (Invitrogen) for 10 min at 37 °C to remove traces of genomic DNA, genomic DNA was treated with RNase I (Invitrogen) for 30 min at 37 °C to remove traces of RNA. RNA and DNA quality and integrity were assessed by standard spectrophotometric and electrophoretic methods, respectively.

### Reverse transcription

RT reactions were carried-out with 1 μg of total RNA using random primers and a High-capacity cDNA Reverse Transcription kit, following manufacturer’s instructions (Applied Biosystems, Foster City, CA).

### Primer design

To amplify baboon *DSCR9* gene and transcript, primers were designed based on highly conserved primate **DSCR9** sequences previously reported [great apes and old world monkeys (OWMs)] and using the online primer-3 tool [10]. Primers (see Table 1) were designed to amplify overlapping target template sequences in order to isolate the complete *DSCR9* gene (in concordance with human gene structure).

**Table 1:** Primers and PCR conditions

**Oligo name**	**Oligo sequence**	**Orientation**	**Primer set**	**Substrate to PCR**	**Initial denatur alization**	**Amplification program**			**Cycles**	**Final elongation**	**Amplicon size**
						**Denaturalization**	**Alignment**	**Elongation**			
OLIGO-rnaF	CTTGGCGCTAAGCTGCCGC	Forware	AMPLICON -mRNA	mRNA	94°/3.5 min	94°/30 seg	60°/45 seg	72°/30 seg	42	72°/15 min	726 pb
OLIGO-rnaR	CCTGCTCTGGAGTCTTGGTG	Reverse									
OLIGO-gene1F	AGCTGGCACTCCCCAGAAT	Forware	OLIGO-gene1	gDNA	94°/5 min	94°/1 min	60°/45 seg	72°/1 min	35	72°/10 min	946 pb
OLIGO-gene1R	GGCTGAGGCACAGAGAAACT	Reverse									
OLIGO-gene2F	CTCCCTACCAAAGTGGCTAG	Forware	OLIGO-gene2	gDNA	94°/5 min	94°/1 min	60°/45 seg	72°/35 seg	30	72°/10 min	769 pb
OLIGO-gene2R	TGTGGAAAGTTGGGGTTTTC	Reverse									
OLIGO-gene3F	GAAAACCCCAACTTTCCACA	Forware	OLIGO-gene3	gDNA	94°/5 min	94°/1 min	60°/45 seg	72°/30 seg	30	72°/10 min	723 pb
OLIGO-gene3R	CCAGGCGAGCAGTCTGTAAC	Reverse									
OLIGO-gene4F	CCTCTCCTGCAACCAATCAG	Forware	OLIGO-gene4	gDNA	94°/5 min	94°/1 min	60°/45 seg	72°/45 seg	30	72°/10 min	807 pb
OLIGO-gene4R	CCCGAATATCCTGGGCTCT	Reverse									
OLIGO-gene5F	CCGGAAGAGCCCAGGATA	Forware	OLIGO-gene5		94°/5 min	94°/1 min	60°/45 seg	72°/1 min	gDNA	35	72°/10 min	967 pb
OLIGO-gene5R	CCGTTTTGGCAGGAATACAT	Reverse										

### PCR amplification

To amplify *DSCR9* gene from genomic DNA five primer sets were used in separate PCR reactions (Table 1). Each PCR reaction was performed in 50 μl reaction containing 10 pM of each primer, 200 ng of genomic DNA and 2X PCR master mix (Qiagen, Valencia, CA). To amplify *DSCR9*-related transcript, a primers set (see Table 1) was used with 10 pM of each primer and 5 μl of RT reaction from each tissue. PCR amplification programs are described in Table 1. Universal 18 s ribosomal gene primers were used in RT-PCR as positive control. (Ambion, Austin, TX). PCR amplifications were confirmed by electrophoresis in agarose gel (1%) run in TAE X1 buffer, stained with etidium bromide and visualized under UV light.

### Molecular cloning and sequencing

PCR products were cloned using the TOPOXL cloning system with the pCR-XL-TOPO 3.5 kb vector (Invitrogen). Ligation reactions were transformed into electrocompetent Top 10 *Escherichia coli* bacterium according to the manufacturer’s instructions (Invitrogen). Cloning products were sequenced in an ABI PRISM 3100 Genetic Analyzer (Applied Biosystems) using universal M13 primers, and Big Dye terminator reagent (Applied Biosystems). Novel sequence has been deposited in the GenBank database (Accession number: JF775469).

### Sequence analysis

Electropherograms were analyzed using GeneStudio Pro software (GeneStudio, Inc., Suwanee, GA). Procedures were carried out in three clones for each amplicon to exclude artifacts. DNA sequences from *DSCR9*-related transcripts were used to determine the amino acid sequence using the Transeq online program [11] and were subsequently aligned using the ClustalW program [12] and Vista tools [13]. The alignments were performed using peptide sequences extracted from GenBank [14] by homology search.

## Results

### Baboon DCSR9 gene isolation

**Figure 1:**
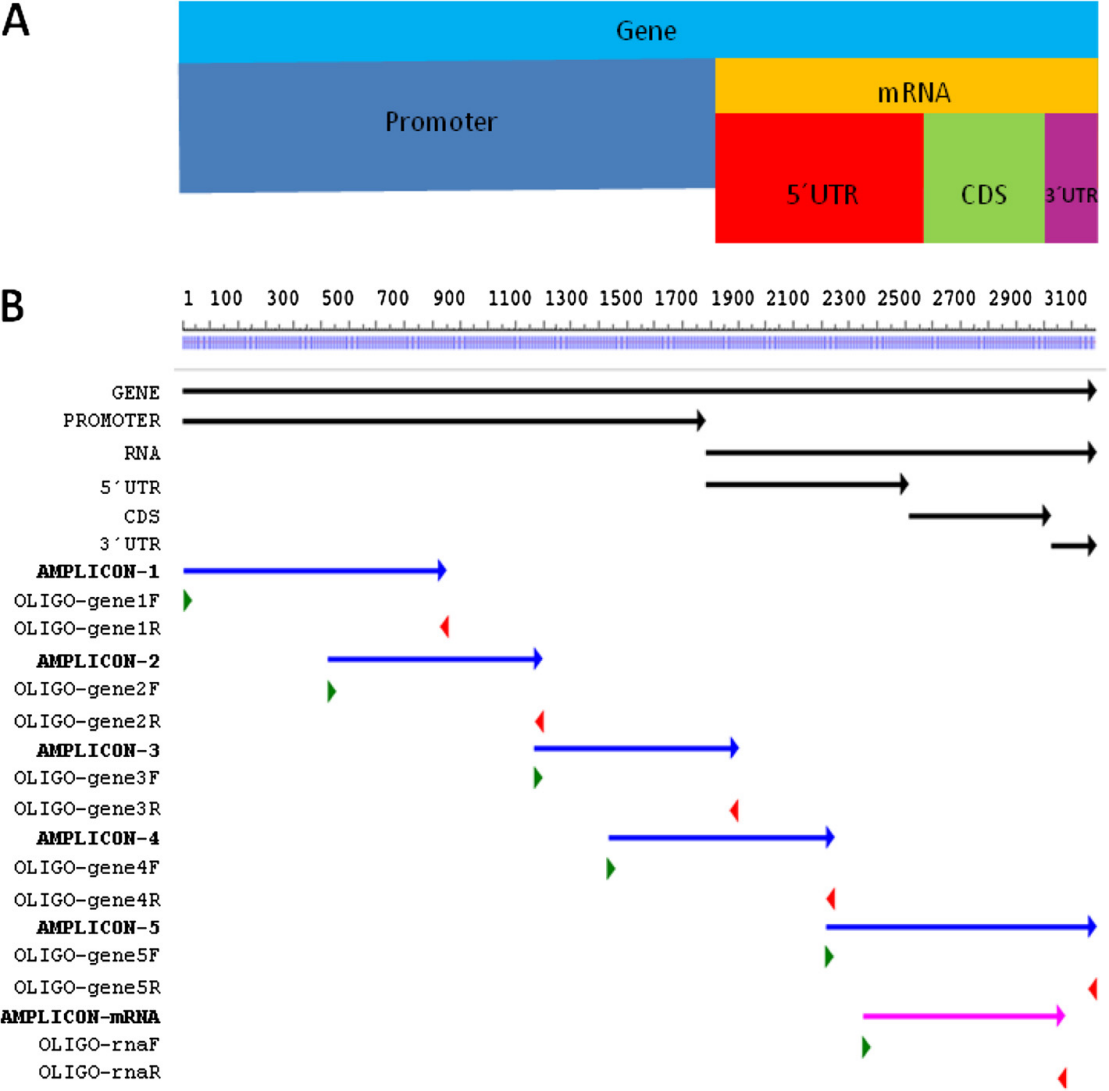
** Strategy to isolate overlapping segments of*Papio hamadryas*DSCR9 gene.** A: Anatomy of the Paha DSCR9 genomic gene. B: Assembly of Paha DSCR9 gene; Primer positions correspond to forward primer and to reverse primers; illustrated amplicons cover the complete DSCR9 amplified gene (blue lines correspond to amplicons generated from genomic DNA and pink line correspond to DSCR9-related transcript).

Baboon genomic *DSCR9* gene was isolated by PCR in five overlapping segments (see Figure 1), which were cloned and sequenced. The assembled sequenced gene was submitted with the BLAST tool of NCBI to the GenBank and match was confirmed with the human *DSCR9* gene (data not shown).

### DSCR9 gene of *papio hamadryas*

**Figure 2:**
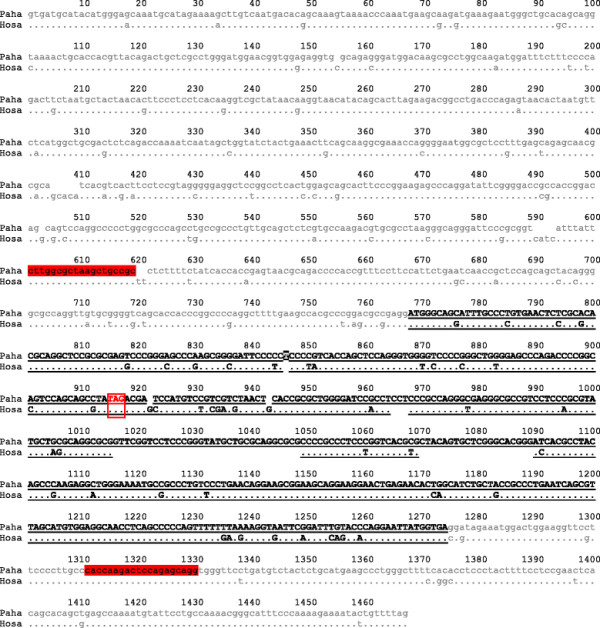
** DSCR9 transcripts.** Nucleotide sequences alignment of baboon and human DSCR9 genes. Both UTRs (5´and 3´) are in grey lower case and the region corresponding to the CDS is in black capital letter. Primers are highlighted in red lower case and the stop codon is in red capital letter

The assembled baboon *DSCR9* gene lacks a TATA box, conventional Kozak sequence [15,16], introns, and polyadenylation signal. The baboon *DSCR9* gene has a structure similar to its human counterpart (Figure 2) but with substantial size difference mainly. For example, while the length of gene, putative promoter, messenger RNA (mRNA), 5´-untranslated region (UTR), coding DNA sequence (CDS) and 3´-UTR for baboon are: 3284, 1861, 1423, 754, 505 and 164 bp (base pair), those of the human are: 3385, 1975, 1410, 767, 450 and 193 bp.

### DSCR9 gene putative promoter analysis

**Figure 3:**
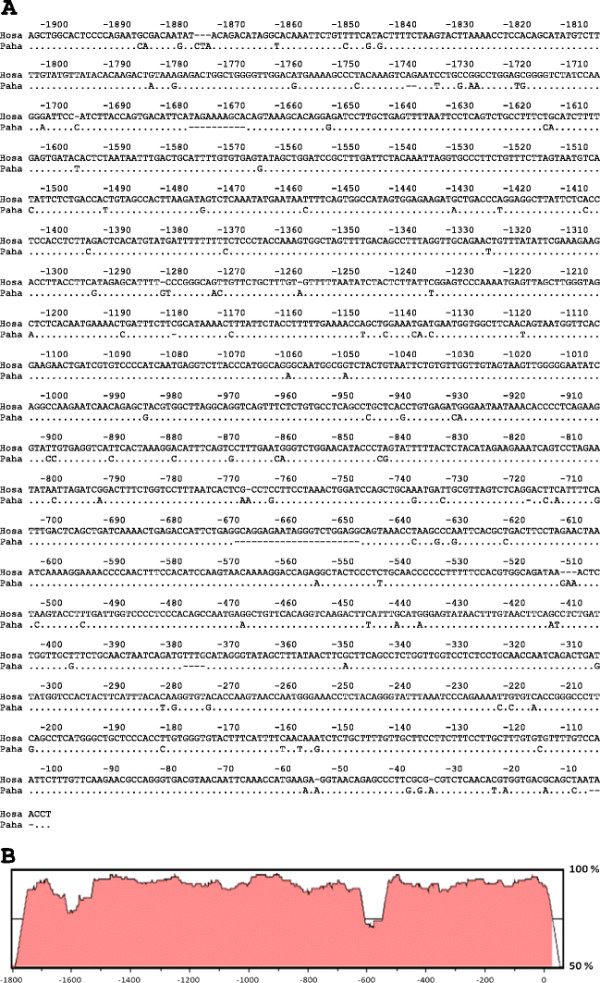
** Comparison of DSCR9 promoter of human vs baboon.** A: sequence alignment. B: Comparison of the two promoters using the Vista Tool program [13].

Based on the structure of the *DSCR9* human gene, we performed the amplification of an upstream region of the transcriptional unit, which we proposed as the putative promoter. The obtained sequence was compared with its counterpart in *Hosa*[9], which was 93% identical (see Figure 3). Additional experiments of transcriptional activity are required to validate the promoter activity of this DNA segment.

### *Papio hamadryas* DSCR9 transcript detection

In order to determine *DSCR9* gene expression in baboon, total RNA was extracted from testis, kidney, cardiac, omental fat, skeletal muscle, pancreas, mononuclear cells, liver, and hypothalamus, placenta and ovary tissues. Total RNA was used for cDNA synthesis by RT. For *DSCR9* transcript isolation an oligo set was designed consisting of a forward consensus primer (DSCR9-rnaF) hybridizing at 148 bases upstream from the translation initiation codon (AUG) and a reverse primer (DSCR9-rnaR) annealing 36 bases after the termination codon (UGA) (positions according to *Hosa DSCR9* mRNA [9]). We detected an amplified product in three different tissues: placental, testis and kidney. A single band was amplified in each case. The size of the amplified product (*Paha*: 726 b) was larger than expected, according to the human sequence (*Hosa*: 673 b). We used amplification of ribosomal RNA 18 s as positive control for each tissue. Negative and positive controls gave the expected results. Expression in heart, omental fat, skeletal muscle, pancreas, mononuclear cells, liver, and hypothalamus was discarded, at least to the sensitivity levels of the used RT-PCR techniques.

The baboon *DSCR9* gene sequence, which is also that of its mRNA since it is an intronless gene, was compared with its *Hosa* counterpart and found to be 90% identical. This value decreases to 89% if just the CDS of both genes are compared. A single thymine nucleotide insertion (underlined in black) results in a premature stop codon (showed in a box), which is predicted to decrease the polypeptide length to 50 aa (see Figure 2). Consequently, from this point on both conceptually translated proteins lose their similarity.

## Discussion

The expression of *DSCR9* gene evidenced in baboon's testicular, kidney and placental tissues coincides with previous reports of expression in humans in the former tissue, but differs in that of the later two organs. The sequences of *DSCR9* gene transcripts in all baboon tissues were identical and without evidence of alternative splicing, unlike those reported for human in which eight different mRNA species have been described (see Table 2). Currently, the predicted DSCR9 protein has not been detected and thus the function of this gene remains unknown. It is hypothesized that allelic variants of the *DSCR9* gene may influence the accumulation of connective tissue of the iris resulting in the phenotype of a different eye color [10]. Probably the *DSCR9* gene is expressed constitutively in the iris and/or acts in a regulatory manner through RNA interference and/or modifies the expression of other genes in the tissue that is associated with a specific phenotype.

**Table 2:** Reported transcripts from nucleotide databases

**Species**	**Tissue**									
	**Testis**	**Unknown**	**Liver**	**Placenta**	**Kidney**	**Skeletal muscle**	**Spleen**	**Brain**	**Caudate nucleus**	**Unknown**
*Homo sapiens*	NR_026719*		AB212291*			AB212287*	AB212 289*	AB212 290*	AK313 458*	
							AB212 288*	AB212 286*		
*Papio anubis*				SRR001694**						
*Macaca fascicularis*	AB168984*				DC640452***					
	CJ490857*									
*Callithrix jacchus*		SRR000079**								SRR000079**

* = Core subset of nucleotide sequence records, ** = SRA, *** = EST of GenBank at the NCBI.

## Conclusion

In our study we found a homologous region to the human *DSCR9* gene in the *Papio hamadryas* genome. We provide evidence of the expression of *DSCR9* gene in baboon's testicular, kidney and placental tissues and of lack of expression in heart, omental fat, skeletal muscle, pancreas, mononuclear cells, liver, and hypothalamus. The segments that compose the transcript unit were assigned based on the strategy described previously by Takamatsu, et al. [9]. Experiments like RNA-RACE are necessary to found 5'-CAP element and designate the length of the 5'-UTR (size in nucleotides). We proposed our gene elements´ nomenclature according to the gene annotation made by Takamatsu et al. [9]. According to our findings, the predicted baboon 5´-UTR has a second ATG sequence that initiates a putative ORF of only three amino acids in length. Then, we agreed with the start codon proposed by Takamatsu et al. [9]. The function of this gene remains unknown, but at least in baboon seems to be dispensable since its corresponding mRNA has a single nucleotide insertion that results in a premature termination of protein. Further studies on the function of this protein in human and other primates are required to understand its role and possible association with the DS phenotype.

## Abbreviations

DS, Down Syndrome; DSCR, Down Syndrome Critical Region; DSCR9, Down Syndrome Critical Region 9; Paha, Papio hamadryas; Hosa, Homo sapiens; b, base; bp, base pairs; PCR, Polymerase Chain Reaction; RT, Reverse Transcription; EST, expressed sequence tag; SRA, Sequence Read Archive; mRNA, messenger RNA; CDS, coding DNA sequence; UTR, untranslated region.

## Competing interests

The authors do not have any potential or actual personal, political, or financial interest in the material, information, or techniques described in this paper.

## Authors’ contributions

IPRS and HABS designed the study. IPRS carried out the main laboratory and bioinformatics work; MLGR aided in analyzing the results and drafted the manuscript; MET, AGC and SAC hosted IPRS for baboon tissue processing. IPRS, MET and HABS drafted the manuscript. All authors read and approved the final manuscript.
